# Direct Thrombin Inhibitor Dabigatran Compromises Pulmonary Endothelial Integrity in a Murine Model of Breast Cancer Metastasis to the Lungs; the Role of Platelets and Inflammation-Associated Haemostasis

**DOI:** 10.3389/fphar.2022.834472

**Published:** 2022-02-28

**Authors:** Marta Smeda, Marta Stojak, Kamil Przyborowski, Magdalena Sternak, Joanna Suraj-Prazmowska, Kamil Kus, Katarzyna Derszniak, Agnieszka Jasztal, Agnieszka Kij, Anna Kurpinska, Anna Kieronska-Rudek, Kamila Wojnar-Lason, Elzbieta Buczek, Tasnim Mohaissen, Stefan Chlopicki

**Affiliations:** ^1^ Jagiellonian Centre for Experimental Therapeutics (JCET), Jagiellonian University, Krakow, Poland; ^2^ Faculty of Chemistry, Jagiellonian University, Krakow, Poland; ^3^ Department of Pharmacology, Jagiellonian University Medical College, Krakow, Poland; ^4^ Faculty of Pharmacy, Jagiellonian University Medical College, Krakow, Poland

**Keywords:** breast cancer, pulmonary metastasis, dabigatran, thrombin, platelets

## Abstract

Activation of the coagulation cascade favours metastatic spread, but antithrombotic therapy might also have detrimental effects on cancer progression. In this study, we characterized the effects of dabigatran, a direct reversible thrombin inhibitor, on the pulmonary endothelial barrier and metastatic spread in a murine model of breast cancer metastasis. Dabigatran etexilate (100 mg kg^−1^) was administered to mice twice daily by oral gavage. Pulmonary metastasis, pulmonary endothelium permeability *in vivo*, and platelet reactivity were evaluated after intravenous injection of 4T1 breast cancer cells into BALB/c mice. The effect of dabigatran on platelet-dependent protection of pulmonary endothelial barrier in the presence of an inflammatory stimulus was also verified *in vitro* using human lung microvascular endothelial cell (HLMVEC) cultures. Dabigatran-treated mice harbored more metastases in their lungs and displayed increased pulmonary endothelium permeability after cancer cell injection. It was not associated with altered lung fibrin deposition, changes in INFγ, or complement activation. In the *in vitro* model of the pulmonary endothelial barrier, dabigatran inhibited platelet-mediated protection of pulmonary endothelium. In a murine model of breast cancer metastasis, dabigatran treatment promoted pulmonary metastasis by the inhibition of platelet-dependent protection of pulmonary endothelial barrier integrity.

## 1 Introduction

The observation that platelet inhibition could hamper cancer metastasis dates back to the 1970s. At that time Gasic et al. (1973), found that acetylsalicylic acid, known as aspirin, compromised metastatic spread. However, despite the mounting evidence that platelets could actively support metastasis through multiple mechanisms (Tesfamariam, 2016), this discovery has not yet resulted in the development or introduction into the clinical setting of an effective anticancer/antimetastatic strategy based on platelet inhibition. In fact, data regarding the beneficial effects of aspirin on cancer progression are conflicting and detrimental effects of antiplatelet therapy on cancer progression were also demonstrated (McNeil et al., 2018; Smeda et al., 2020a).

In recent years, the idea to hamper cancer metastasis via anticoagulant/antiplatelet therapy has re-emerged due to the development of novel potent oral anticoagulants (NOACs), which affect pathways of platelet activation and thrombus formation that are independent of cyclooxygenase 1-dependent thromboxane A2 production, a pathway inhibited by aspirin. Although NOACs were shown to reduce cancer metastasis (DeFeo et al., 2010; Alexander et al., 2015) several studies also provide evidence for the lack of antimetastatic effects of these compounds (Alexander et al., 2015; Buijs et al., 2019), and some even suggest detrimental effects (Niers et al., 2009; Shi et al., 2017). The inconsistency regarding anticancer/antimetastatic effects also applies to the direct reversible thrombin inhibitor dabigatran. Although dabigatran effectively inhibited metastatic spread in some studies (DeFeo et al., 2010), in another studies it was ineffective (Alexander et al., 2015; Buijs et al., 2019), or it increased metastatic seeding (Shi et al., 2017).

Since the mechanism of possible pro-metastatic effects of dabigatran has not yet been resolved, this study aimed to investigate whether the effect of dabigatran increasing the formation of pulmonary metastases could be linked to alterations in pulmonary endothelial barrier permeability after intravenous (i.v.) injection of 4T1 breast cancer cells, which are known to trigger an inflammatory response in the lungs (Häuselmann et al., 2016) associated with mechanisms of inflammation-associated haemostasis (Ho-Tin-Noé et al., 2018). In our study, we suggest that the mechanisms underlying platelet-dependent inflammation-associated haemostasis might afford protection to the endothelial barrier in cancer metastasis, and their inhibition might indeed unfavourably determine the outcome of antiplatelet therapy on cancer metastasis, a phenomenon that has not been previously appreciated.

## 2 Materials and Methods

### 2.1 Animals

Female BALB/c mice (aged 7–11 weeks, 140 mice) were purchased from the Medical University of Bialystok (Poland) and housed 5–6 per cage in a temperature-controlled environment (22–25°C), 12-hour light/day cycle, and unlimited access to food (Zoolab, Krakow, Poland) and water throughout the experiment. All experimental procedures involving animals were accomplished in accordance with the approval of the Second Local Ethical Committee on Animal Testing, permit no 91/2018 and 259/2019. Mice welfare was monitored throughout the study once daily. Euthanasia was performed by intraperitoneal (i.p.) injection of ketamine and xylazine, 100 and 10 mg ⋅ kg^−1^ of body weight, respectively.

### 2.2 Cell Culture

*In vivo* studies utilised the mouse mammary adenocarcinoma 4T1-luc2-tdTomato cell line stably expressing the firefly luciferase gene and tdTomato fluorescent protein (the kind gift of Professor Joanna Wietrzyk from Ludwik Hirszfeld Institute of Immunology and Experimental Therapy, Polish Academy of Sciences (IIET)) at the 5^th^ passage from resuscitation after their purchase from Caliper Life Sciences Inc., USA; source of the parental line: ATCC, CRL-2539). The cells were cultured as previously described (Smeda et al., 2020a). Prior to i.v. injection, the cells were detached using Accutase solution (Sigma-Aldrich, Poland), centrifuged (300 × g, 4°C, 5 min), stained with Cell Tracker™ Red CMTPX Dye (Invitrogen, C34552) for 30 min at 37°C, rinsed 3 times with Dulbecco’s phosphate-buffered saline (DPBS) (Gibco), DPBS and growth medium 1:1 (Hank’s Balanced Salt Solution (HBSS), IIET, Poland), resuspended in HBSS at the required concentration, and injected into the tail vein of female BALB/c mice (7.5 × 10^4^ cells in 100 μl of HBSS per mouse). For *in vitro* experiments, the HLMVEC line was used. The cell line was obtained from the European Cell Culture Collection (Cell Applications), cultured in microvascular endothelial cell growth medium (Cell Applications) at 37°C in an atmosphere of 5% CO_2_. Both cell cultures were routinely tested for *Mycoplasma* contamination.

### 2.3 Pulmonary Endothelium Permeability *In Vivo*

Anaesthetized mice (100 mg ⋅ kg^−1^ ketamine +10 mg ⋅ kg^−1^ xylazine, i.p. were injected via the femoral vein with a solution of 2% Evans blue (EB; 60 kDa) dye (Sigma Aldrich) in .9% saline at a dose of 4 mg ⋅ kg^−1^, as described previously (Smeda et al., 2018). The injected dye solution was left to circulate for 10 min, and the lungs were perfused with PBS for 15 min, isolated, weighted, and homogenised in 200 μl of 50% TCA (dissolved in distilled water). The homogenate was centrifuged (at 12,000 rpm for 12 min at 4°C) and stored at −80°C. Prior to EB concentration measurement (absorbance at 620 nm, plate reader Synergy 4, BioTek), the supernatant was diluted at a 1:3 ratio with 95% ethanol. The results were normalised to tissue weight ***.***

### 2.4 Assessment of Pulmonary Metastasis

Excised lungs were washed with saline, fixed in formalin, paraffin-embedded, and cut into 5-μm slices. For the 24 h- and 2-day time points, the number of viable cancer cell colonies was counted based on their vivid and stable red fluorescence and normalised to the area of the lung cross-section. For the 7-day time point, quantitative assessment of already well-defined pulmonary metastases was based on classical haematoxylin and eosin (H&E) staining and normalised to the area of the lung cross-section as described previously (Smeda et al., 2020a).

### 2.5 Effects of Dabigatran on Metastatic Seeding Into the Lungs in the Murine Model of Experimental Metastasis

Thirty mice were pre-treated with dabigatran etexilate (Biorbyt, cat. no orb180748) by oral gavage twice daily at a total dose of 100 mg per kg of body weight per day in .05% natrosol, and the same number of mice simultaneously received the vehicle (.05% natrosol) for 3 consecutive days. The dosing regimen effectively reduced pulmonary metastasis in the orthotopic model of breast cancer (DeFeo et al., 2010). All mice were subsequently injected with 7.5 × 10^4^ mouse mammary adenocarcinoma 4T1-luc2-tdTomato (4T1) cells. Dabigatran etexilate or vehicle treatment was continued (twice daily Monday to Friday and once daily over the weekend) until the animal euthanasia at 24 h, 2 days, and 7 days after injection of cancer cells.

### 2.6 Effects of Dabigatran on Pulmonary Permeability in a Murine Model of Experimental Metastasis

To measure pulmonary permeability alongside the progression of cancer, 40 mice were pre-treated with dabigatran etexilate in .05% natrosol twice daily at the final dose of 100 mg ⋅ kg^−1^ 24 h, while the other 40 received the vehicle (.05% natrosol). Afterwards, sixty mice were injected with 4T1 cells and euthanized at 24-h, 2-day, and 7-day timepoints after the injection, whereas 20 healthy control mice were euthanised after the pre-treatment period to visualise if dabigatran etexilate affected the pulmonary endothelium barrier in the mice not injected with breast cancer cells. Just before euthanasia, all animals were injected intravenously with EB dye solution, perfused with phosphate-buffered saline, and their lungs were excised for subsequent analysis.

### 2.7 Immunohistochemical Staining

Lungs were fixed in formalin, paraffin-embedded, cut into 5-μm slices, and mounted on the slides. For fibrinogen immunohistochemical staining, deparaffinized slide-attached lung cross-sections were stained with polyclonal goat antiserum to mouse fibrin(ogen) 1:250 (Accurate Chemical & Scientific Corporation, YNGMFBGBio), followed by biotin-SP donkey anti-goat secondary antibody 1:600 (Jackson Immuno Research, 112-065-003). Stained lung cross-sections were subsequently scanned with a BX51 microscope equipped with virtual microscopy system dotSlide (Olympus, Japan). Image segmentation was performed in Ilastik (developed by the Ilastik team, with partial financial support by the Heidelberg Collaboratory for Image Processing, HHMI Janelia Farm Research Campus, and CellNetworks Excellence Cluster).

### 2.8 Calibrated Automated Thrombography

The effect of dabigatran on thrombin activity was measured in murine plasma using a thrombin generation assay according to (Tchaikovski et al., 2007) with major modifications. First, citrated murine plasma was mixed with fluorogenic substrate (Z-Gly-Gly-Arg-AMC) solution and pipetted into the plate wells, and thrombin generation was initiated by the addition of the trigger solution, containing tissue factor (TF), phospholipids (PL), and CaCl_2_. As a result, 60 µl of the prepared mixture in a well contained 12 µl of plasma, 9 µl of substrate solution, and 39 µl of trigger solution, with a final concentration of 20% plasma, 1.0 pM TF, 16.25 mM CaCl_2_, 4 μM PL, and .43 mM ZGGR-AMC. Each plasma sample was calibrated by replacing the trigger solution with a solution containing α2-macroglobulin-thrombin complex (α2M-T, at a final concentration corresponding with 44 nM thrombin activity). Measurements were performed at 37°C and each sample was tested in duplicate. Fluorescent signals were recorded using Tecan Spark 10M microplate reader (Männedorf, Switzerland) and transformed into thrombin concentration as described previously (Hemker and Kremers, 2013). The effect of dabigatran was evaluated based on the parameter lag time, representing the time required to start the generation of thrombin. All reagents were provided as a gift by Synapse BV, Netherlands.

### 2.9 Determination of Protein Level/Concentration in Lung Homogenates

Lungs were excised, rinsed in saline, dried with tissue paper, weighed, cut into small pieces, and snap-frozen in liquid nitrogen; the samples were stored at − 80°C. Lung tissue was homogenised in T-PER buffer (Thermo Scientific, 78510), total protein concentration was determined with BCA Assay Kit (Thermo Scientific, 23225), and the homogenates were aliquoted and stored at −80°C for further analysis. Concentrations of INFγ and iC3b were determined with commercially available ELISA kits (R&D Systems (MIF00) and MyBioSource (MBS9393088), respectively). For Western blotting, an equal amount of protein was loaded and run on the gels, and then transferred to a nitrocellulose membrane, blocked overnight with 5% dry milk, and incubated overnight with the appropriate primary antibodies in TBS (Tris-buffered saline) directed against the following antigens: Ang-2 (Thermo Scientific, PA5-27297, 1:10 000), E-selectin (Bioss Antibodies, bs-1273R, 1:1000), MMP-9 (Millipore, AB19016, 1:1000). After washing, membranes were incubated with anti-rabbit secondary antibodies conjugated with HRP (Santa Cruz Biotechnology, sc-2357, 1:5000) diluted in TBS buffer. Chemiluminescent signal was developed with Clarity Western ECL Substrate (Bio-Rad, 1705061) using ChemiDoc Imager (Bio-Rad) and densitometric analysis of band intensity was performed in ImageJ. The total protein loaded onto the particular lane after transfer was used as the loading control using stain-free technology from Bio-Rad as previously described (Smeda et al., 2018).

### 2.10 Platelet Reactivity to a Low Dose of Thrombin

Mouse citrated blood (blood/3.8% citrate at 10:1 (v/v)) was diluted with saline and washed with Tyrode’s buffer. Washed blood samples obtained from dabigatran-treated or untreated cancer cell-injected mice were directly subjected to thrombin (.025 U ml^−1^) activation and antibody staining described below, whereas washed blood samples derived from healthy mice were pre-incubated with 1, 10, 30, and 100 ng ml^−1^ of dabigatran or dabigatran solvent alone (DMSO; the final DMSO concentration was <1%) to assess the effects of the given dabigatran concentrations on platelet thrombin-induced (.025 U ml^−1^ or .1 U ml^−1^) reactivity. Each blood sample was double-stained with an antibody panel that included a platelet-specific antigen, FITC-conjugated GpIIb/IIIa (CD41/61) (Rat IgG2a, clone Leo.F2, Emfret Analytics, #M025-1, 1:5) for platelet identification as well as a platelet activation marker the PE-conjugated active form of GPIIb/IIIa (Rat IgG2b, clone: JON/A, Emfret Analytics, M023-2, 1:5). Platelets were identified based on their forward- and side-scatter characteristics and gated based on the expression of platelet-specific antigen CD41/61 as previously described (Smeda et al., 2018). Unstained platelets were used to establish the level of autofluorescence that was set to fall within the first log order of brightness for each fluorescence channel. FITC-conjugated isotype control antibody (Rat IgG (negative control), Emfret Analytics, P190-1, 1:5) was used to assess non-specific binding for each individual sample. Basal and thrombin-induced (.025 U ml^−1^ or .1 U ml^−1^) reactivity of circulating platelets was assessed based on the measured expression/binding level of an active form of GPIIb/IIIa expressed as the percentage of all platelets above isotype control fluorescent signal and/or the median fluorescence intensity (MFI). Flow cytometric analyses of platelet activation was performed using BD LSR II instrument (BD Biosciences) and analysed in BD FACSDiva 6.0 software (Becton Dickinson, Oxford, United Kingdom). Measurements were made on a logarithmic scale and at least 10,000 events were collected for each sample. Appropriate colour compensation was determined in samples singly stained with FITC-conjugated anti-CD41/61. FITC and PE dyes were excited by a 488 nm laser.

### 2.11 Measurement of Plasma Dabigatran Concentration

The concentration of dabigatran (administered to mice as a pro-drug dabigatran etexilate) was assessed in plasma samples using the UHPLC-MS/MS technique. The plasma volume of 50 µl was spiked with 5 µl of internal standard (IS, dabigatran-^13^C_6_) at the concentration of 1 µg ml^−1^. After gently shaking, 150 µl of .1 M HCl in methanol was added, mixed for 10 min and chilled at 4°C for 10 min. The supernatant collected after sample centrifugation (15,000 rpm, 4°C, 15 min) was directly injected onto an UltiMate 3000 UHPLC system (Thermo Fisher Scientific, Waltham, MA, United States) combined with a TSQ Quantum Ultra triple quadrupole mass spectrometer (Thermo Fisher Scientific, Waltham, MA, United States). The chromatographic analysis was conducted using an ACQUITY UHPLC BEH C18 (3.0 × 100 mm, 1.7 μm, Waters, Milford, MD, United States) analytical column and applying .1% FA in ACN (A) and .1% FA in H_2_O (B) as mobile phases delivered in the following gradient elution program: 95% B for 1 min, 95%–5% B for 3 min, 5%–95% B for .5 min, and 95% B for 2.5 min for column equilibration. The mass spectrometric detection was conducted in an electrospray positive ionization mode, and selected ion transitions were used for quantification: 472.4→172.0 (CE = 39 V) and 478.3→172.1 (CE = 39 V) for dabigatran and dabigatran-^13^C_6_, respectively. The plasma concentration of dabigatran was calculated based on the regression equation determined for the calibration curve plotted for dabigatran and expressed as the relationship between the peak area ratios of analyte/IS to the nominal concentration of the analyte.

### 2.12 Pulmonary Endothelium Permeability *In Vitro*

The response of the barrier formed by human lung microvascular endothelial cells (HLMVECs) to an inflammatory agent (IL-1β, 10 ng ml^−1^) was assessed in real-time in a fully standardized manner by continuously recording changes in electrical resistance with an ECIS system (Bernas et al., 2010), using 8W10E + or 96W10E + electrode chamber arrays and an ECIS Z-Theta system (Applied Biophysics), along with the associated software v.1.2.126 PC. Immediately after cell seeding, resistance measurements (in Ohms) were initiated, and the increase in resistance with respect to time indicated that cells were forming contacts between each other. The steady state was achieved when maximum resistance was reached, which indicated the formation of a tight monolayer.

On that day, platelets were isolated from the human citrate blood samples obtained from the Blood Donation Centre. Obtained platelet-rich plasma (PRP) (blood was centrifuged at 260 × g with slow deceleration) was supplemented with prostacyclin to a final concentration of 100 ng ml^−1^ and centrifuged to obtain platelet pellets (960 × g for 10 min). Platelet pellets were resuspended in PBS with albumin (1 mg ml^−1^) and glucose (1 mg ml^−1^), subsequently centrifuged in presence of prostacyclin (100 ng ml^−1^, 810 × g for 10 min), and then the supernatant was discarded. Washed platelets were resuspended in Tyrode’s buffer to a final number of 1 × 10^6^ PLT and stimulated with bovine thrombin (.1 U ml^−1^), collagen (5 μg ml^−1^), or ADP (20 μM) or remained unstimulated for 15 min, including their centrifugation time (10 min, 900 × g). Some platelet samples were pre-treated with dabigatran (30 ng ml^−1^) and subsequently stimulated with bovine thrombin (.1 U ml^−1^) or remained unstimulated. After centrifugation to discard platelets, platelet releasates were added to HLMVEC cultures with or without IL-1β, and Tyrode’s buffer was added to control wells. Changes in cell resistance were measured in real-time for the consecutive 75 min.

### 2.13 Biomarkers of Endothelial Dysfunction

Measurement of the plasma concentration of selected endothelial dysfunction markers was performed using the microLC/MS-MRM method as described previously (Walczak et al., 2015; Suraj et al., 2018; Suraj et al., 2019a; Suraj et al., 2019b). The panel used in this study included biomarkers of endothelium permeability (Ang-1, Ang-2, sFLT-1 (soluble form of fms-like tyrosine kinase 1), and sTie-2 receptor), biomarker of glycocalyx disruption (SDC-1), biomarkers of endothelium inflammation and haemostasis (sE-sel, sICAM-1, vWF, t-PA, and PAI-1), and biomarkers of platelet activation (sP-sel and THBS-1). To assess the plasma concentration of the selected biomarkers, the UHPLC Nexera system (Shimadzu, Kyoto, Japan) connected with a highly sensitive mass spectrometer, QTrap 5500 (Sciex, Framingham, MA, United States), were used. Sample preparation included proteolytic digestion with porcine trypsin to achieve the unique and reproducible peptide sequences that were applied as the surrogates of the proteins suitable for LC-MS/MS analyses. A detailed description of the targeted analysis of the selected panel of proteins was presented elsewhere (Suraj et al., 2018; Suraj et al., 2019a; Suraj et al., 2019b).

In brief, total protein concentration in plasma was assessed using a NanoDrop 8000 spectrophotometer (Thermo Fisher Scientific, Waltham, MA, United States). The samples were diluted to a concentration of 7 mg ml^−1^ using 25 mM ammonium bicarbonate (NH_4_HCO_3_) (Sigma-Aldrich) and 210 μg of total protein was denatured with 10% solution of sodium deoxycholate (Sigma-Aldrich) in 25 mM NH_4_HCO_3_, then diluted with 25 mM NH_4_HCO_3_. Samples were reduced with 50 mM tris(2-carboxyethyl)phosphine (TCEP) (Sigma-Aldrich) in 25 mM NH_4_HCO_3_ for 30 min at 60°C and alkylated in the dark with 100 mM iodoacetamide (IAM) (Sigma-Aldrich) in 25 mM NH_4_HCO_3_ for 30 min at 37°C. Next, the excess of IAM was quenched by the addition of 100 mM DL-dithiothreitol (DTT) (Sigma-Aldrich) in 25 mM NH_4_HCO_3_. The incubation of the samples lasted 30 min at 37°C. Proteolytic digestion was carried out for 16 h at 37°C with sequencing grade modified trypsin (400 µg ml^−1^) in a 50:1 ratio (substrate:enzyme) (Promega, Madison, USA). Prepared internal standard peptide solutions obtained from dissolved powders of internal standard peptides, synthesized and quality-controlled by Innovagen (Lund, Sweden) (Suraj et al., 2018; Suraj et al., 2019b); specific for each target peptide sequence were implemented just before ceasing the digestion. Finally, this process was stopped by the addition of formic acid (FA) (Sigma-Aldrich) at a final concentration of .5% v/v. Next, sodium deoxycholate was pelleted. The sample supernatant obtained after centrifugation (3,000 × g for 10 min at 23°C) was desalted and concentrated by the micro-solid phase extraction (μSPE) procedure using the Oasis HLB elution plate with 2 mg of sorbent mass per well (Waters, Milford, USA). Finally prepared samples were lyophilized and, directly before LC-MS/MS analyses, resuspended in 50 μl 20% ACN in H_2_O.

### 2.14 Statistical Analysis

Statistical significance of *in vivo* data was assessed in GraphPad Prism 5.03 with a parametric two-way ANOVA or non-parametric Kruskal-Wallis test followed by the appropriate *post hoc* test (Bonferroni or Dunns Multiple Comparison Tests) based on the normality of the data distribution (tested with a Shapiro-Wilk normality test), homogeneity of variances (tested with an F test), and the variable scale. The data were presented as the median and the interquartile range (IQR) (from lower (25%) to upper (75%) quartile). Only *p* values ≤.05 were considered significant. We analysed the differences between untreated mice at given timepoints (black symbols on the graphs), the differences between dabigatran-treated mice at given timepoints (grey symbols on the graphs), and the differences between untreated and dabigatran-treated mice at the given timepoint, and significant differences (*p* < .05) were indicated in the graphs. The significant outliers identified by Grubbs test were excluded from the statistical analysis. The *in vitro* data illustrating changes of the endothelial barrier in real time were subjected to the analysis of covariance (ANCOVA) based on their parallel course confirmed with the model of equal slopes analysis in Statistica 13.

## 3 Results

### 3.1 Effects of Dabigatran Treatment on Metastatic Spread Into the Lungs of BALB/c Mice Injected With 4T1 Breast Cancer Cells

**FIGURE 1:**
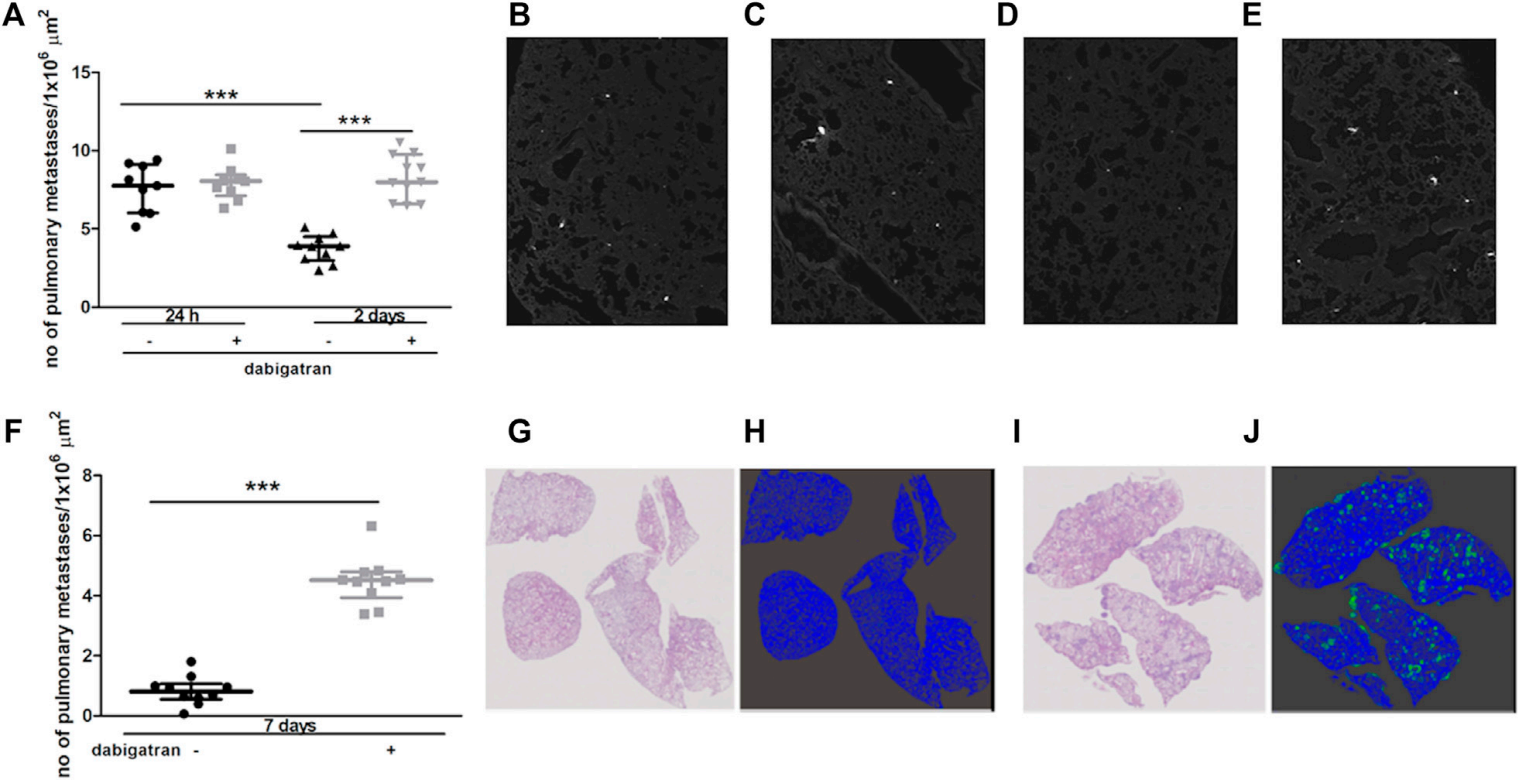
Effects of dabigatran on pulmonary metastasis of 4T1 breast cancer cells injected intravenously into BALB/c mice. Mice were treated with vehicle (black symbols) or dabigatran etexilate (grey symbols) as described in Section 2.5, injected with 4T1 breast cancer cells, and euthanized 24 h, 2 days, and 7 days after injection. In **(A)** quantitative analysis of pulmonary metastasis in mice, based on CellTracker Red fluorescence in murine lungs isolated from animals 24 h and 2 days after injection of 4T1 cancer cells, is shown. The representative pictures of lung parenchyma illustrating metastatic count in untreated mice 24 h and 2 days after i.v. injection (100 x) are shown in **(B,D)**, respectively; the representative images of dabigatran-treated mice 24 h and 2 days after injection are shown in **(C,E)**, respectively. In **(F)**, quantitative analysis of pulmonary metastasis based on haematoxylin and eosin (H&E) staining 7 days after i.v. injection is shown. The representative images of H&E-stained lung cross-sections (100×) of mice not treated and treated with dabigatran etexilate are shown in **(G,I)**, respectively, while the results of Ilastik segmentation of **(G,I)** are shown in **(H,J)** (blue: lung parenchyma, green: pulmonary metastases), respectively. Statistical analysis in **(A)** was performed using a two-way ANOVA test followed by Bonferroni post hoc tests, while the results in **(F)** were analysed with two-sided unpaired T test. The results are presented as the median ± IQR. The symbol *** denotes statistical significance at *p* < .001.

Intravenous injection of 4T1 breast cancer cells into female BALB/c mice resulted in robust accumulation of cancer cells in their lungs. The number of metastatic cancer cells was high 24 h after 4T1 cancer cell injection (Figures 1A,B) and then progressively declined on the 2^nd^ and 7^th^ day after cancer cell injection (Figures 1A,D,F–H). These findings could be explained by the progressive apoptosis of intravascular cancer cells that did not form metastatic colonies, as shown previously by (Qiu et al., 2003). In the lungs of dabigatran-treated mice, the number of accumulated cancer cells was not different than that in the lungs of untreated mice 24 h after the injection (Figures 1A–C); however, on the 2^nd^ and 7^th^ day after 4T1 cancer cell i.v. administration, the number of early cancer cell colonies and well-established pulmonary metastases, respectively, were substantially higher in dabigatran-treated mice than in the untreated mice (Figures 1A,D–J).

### 3.2 Effects of Dabigatran Treatment on Pulmonary Fibrin Deposition, Plasma Thrombin Generation, and Activation of the Innate Immune System Alongside the Development of Metastasis in BALB/c Mice Injected With 4T1 Breast Cancer Cells

**FIGURE 2:**
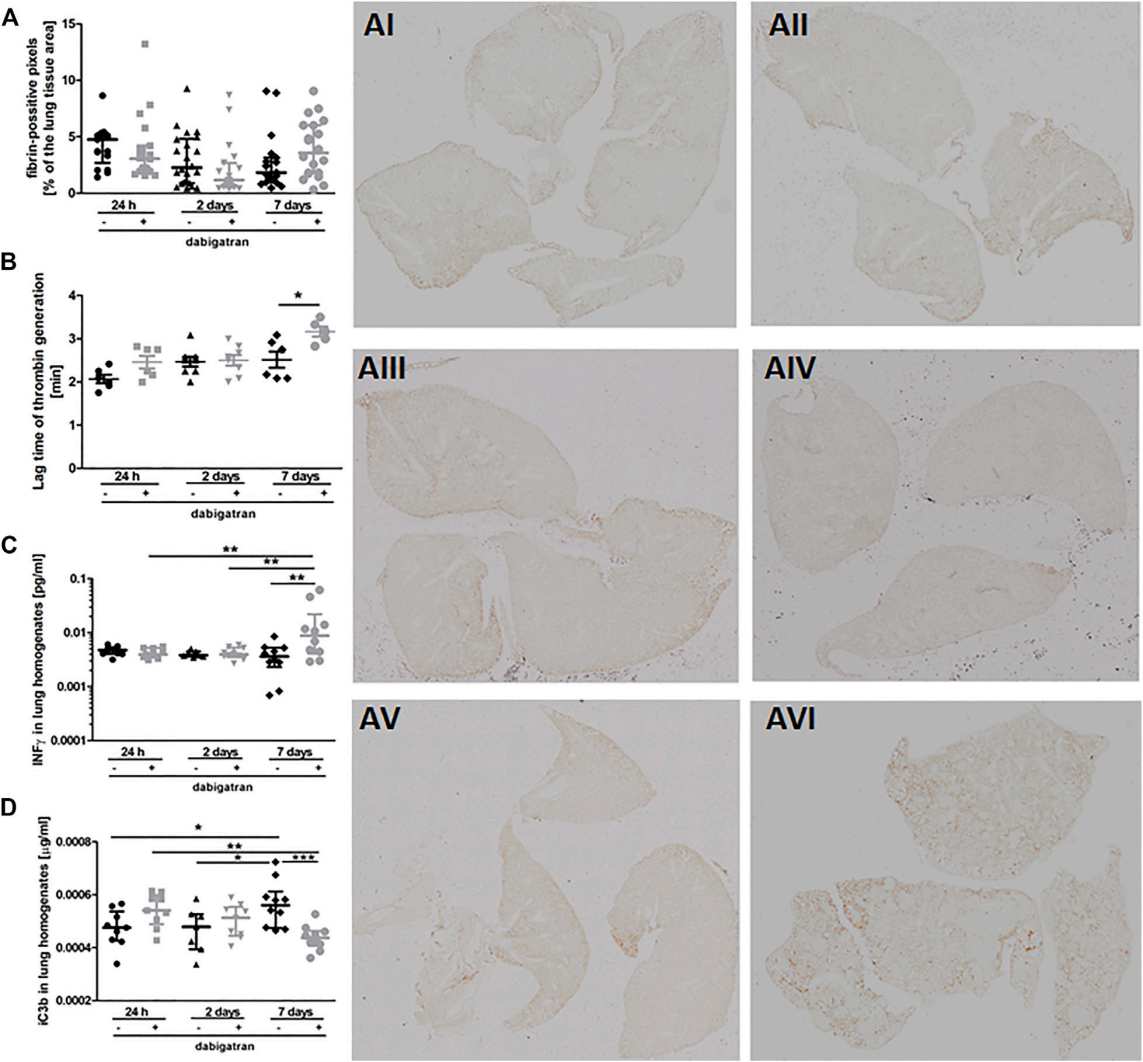
Pulmonary fibrin deposition, lag time of thrombin generation in plasma, and innate immunity activation markers. Mice were treated with vehicle (black symbols) or dabigatran etexilate (grey symbols) as described in Section 2.5, injected with 4T1 breast cancer cells, and euthanized at 24 h, 2 days, and 7 days after injection. **(A)** Lung cross-sections were stained with biotin-conjugated IgG fraction of polyclonal goat antiserum to mouse fibrin antibody as described in Section 2. The representative images of fibrin deposition in the lungs of 4T1 breast cancer cell-injected mice are given in AI (untreated, 24 h post i.v.), AII (dabigatran-treated, 24 h post i.v.), AIII (untreated, 2 days post i.v.), AIV (dabigatran-treated, 2 days post i.v.), AV (untreated, 7 days post i.v.), AVI (dabigatran-treated, 7 days post i.v.) (200x). Scanned images were segmented using Ilastik software and counted in ImageJ to determine the number of pixels corresponding to fibrin signal; **(B)** the thrombin activity was determined by thrombin generation assay in murine plasma, and expressed as a lag time to cleavage of fluorogenic substrate by free thrombin; **(C)** concentrations of interferon γ (IFNγ) and **(D)** complement iC3b subunit were determined in lung homogenates by ELISA kits. Statistical analysis was performed with Kruskal-Wallis **(A)** and two-way ANOVA **(B–D)** tests followed by appropriate *post hoc* tests, and the results were presented as median ± IQR. The symbols *, **, and *** denote statistical significance at *p* < .05, *p* < .01, and *p* < 0.001, respectively.

To verify whether the unfavourable effects of dabigatran on pulmonary metastasis could be related to inhibition of fibrin deposition or innate immune response, we quantitively analysed fibrin levels in the murine lungs (Figure 2A) in relation to thrombin generation in plasma (Figure 2B), interferon γ (IFNγ) concentration in lung homogenates (Figure 2C), and complement activation (Figure 2D). Dabigatran treatment of 4T1 breast cancer cell-injected mice did not affect fibrin deposition in the lungs (Figure 2A). At early timepoints (24 h and 2 days after injection), dabigatran treatment also did not affect the lag time of thrombin generation in the plasma (Figure 2B) (though it tended to be increased 24 h after injection for dabigatran-treated mice), did not alter the concentration of IFNγ reflecting activation of NK cells (Takeda et al., 2011) (Figure 2C), or did not modify the activation of complement (Figure 2D). The changes in the above parameters observed on the 7^th^ day after i.v. injection were rather secondary and might have resulted from the difference in the metastatic count at that time between untreated and dabigatran-treated mice (Figure 1F).

### 3.3 Effects of Dabigatran Treatment on Lung Permeability and Expression of Inflammatory Markers in the Lung Parenchyma Alongside the Development of Metastasis in BALB/c Mice Injected With 4T1 Breast Cancer Cells

**FIGURE 3:**
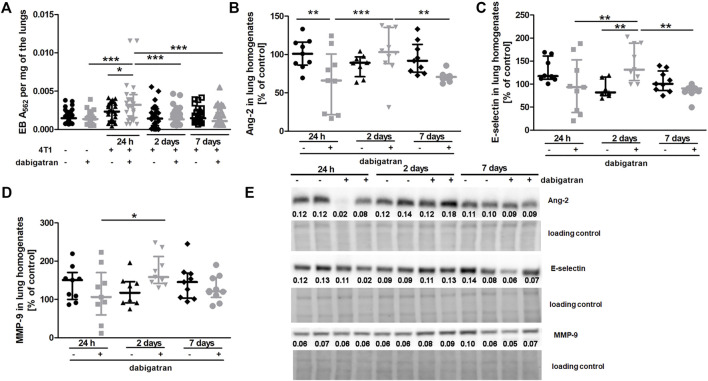
Effects of dabigatran on lung permeability and inflammation markers in the lungs of BALB/c mice injected with 4T1 breast cancer cells. Mice were treated with vehicle (black symbols) or dabigatran etexilate (grey symbols) as described in Section 2.5. **(A)** depicts retention of Evans blue (EB) in the lung parenchyma, reflecting lung permeability. Control mice did not receive cancer cells and were euthanized at the end of the dabigatran pre-treatment to exclude the possibility that dabigatran affected pulmonary permeability of healthy animals before their inoculation with 4T1 cancer cells. After dabigatran/vehicle pre-treatment, the other mice were injected intravenously with 4T1 breast cancer cells or the vehicle and euthanized 24 h, 2 days, and 7 days after injection. **(B–D)** show the levels of Ang-2, E-selectin, and MMP-9, respectively. **(E)** shows representative Western blot images of Ang-2, E-selectin, and MMP-9 with densitometric data after their normalization to the total protein used as loading control based on the stain-free technique as described in *Materials and Methods*. For **(A–D)**, statistical analysis was performed with two-way ANOVA and an appropriate post hoc test. The symbols *, **, and *** denote statistical significance at *p* < .05, *p* < .01, and *p* < .001, respectively, and the data are depicted as the median ± IQR.

Treatment with dabigatran did not alter lung permeability of mice not injected with 4T1 breast cancer cells (Figure 3A). Surprisingly, 4T1 cancer cell injection resulted in increased pulmonary vascular permeability in dabigatran-treated mice 24 h after cancer cells injection (Figure 3A). The higher lung permeability 24 h after i.v. injection in dabigatran-treated and 4T1 cancer cell-injected mice was followed by increased breast cancer cell extravasation and settlement in the lungs 2 days after injection (Figure 1A) that was associated with an increased inflammatory response in the lungs of dabigatran-treated mice (Angiopoietin-2 (Ang-2), Figure 3B; E-selectin, Figure 3C; and matrix metalloproteinase 9 (MMP-9), Figure 3D).

### 3.4 Effects of Dabigatran Treatment on the Panel of Endothelial Dysfunction Biomarkers in the plasma, along with the Development of Metastasis in BALB/c Mice Injected With 4T1 Breast Cancer Cells

**FIGURE 4:**
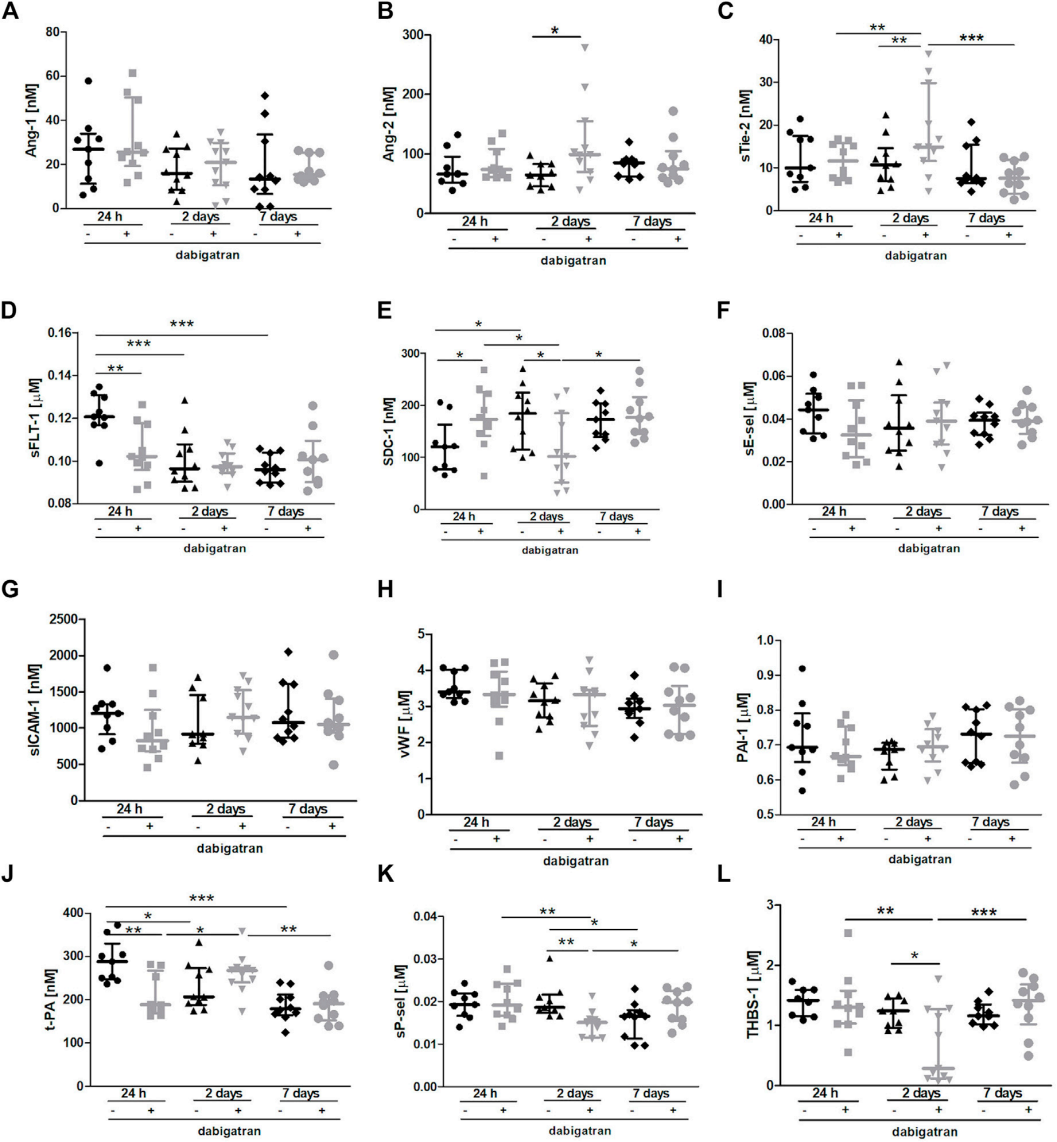
Effects of dabigatran on endothelium function based on selected biomarkers of endothelial dysfunction in BALB/c mice injected with 4T1 breast cancer cells. Mice were treated with vehicle (black symbols) or dabigatran etexilate (grey symbols) as described in Section 2.5, injected with 4T1 breast cancer cells, and euthanized at 24 h, 2 days, and 7 days after 4T1 breast cancer cell injection. The panel of selected biomarkers of endothelial dysfunction (Ang-1 **(A)**, Ang-2 **(B)**, sTie-2 **(C)**, sFLT-1 **(D)**, SDC-1 **(E)**, sE-sel **(F)**, sICAM-1 **(G)**, vWF **(H)**, PAI-1 **(I)**, t-PA **(J)**, sP-sel **(K)**, and THBS-1 **(L)**) was measured in the plasma using the microLC/MS-MRM method as described in Section 2. The data are presented as the median ± IQR. Statistical analysis was performed with two-way ANOVA **(A,C–H,J–L)** or Kruskal-Wallis **(B,I)** tests followed by appropriate *post hoc* tests. The symbols *, **, and *** denote statistical significance at *p* < .05, *p* < .01, and *p* < .001, respectively.

The Figure 4 shows selected plasma parameters that might indicate systemic endothelial dysfunction. To assess the functional status of the endothelium at the systemic level in the absence or presence of dabigatran treatment in 4T1 breast cancer cell-injected mice, selected protein biomarkers of endothelial dysfunction were measured in the mouse plasma (Figure 4) as previously described in the orthotopic 4T1 breast cancer model (Suraj et al., 2019a). The panel used in this study included selected biomarkers of endothelial permeability (Angiopoietin 1 (Ang-1), Ang-2, soluble form of Tie-2 (sTie-2), and soluble form of VEGF receptor 1 (sFLT-1); Figures 4A–D, respectively), glycocalyx damage (syndecan 1 (SDC-1); Figure 4E), endothelial inflammation and haemostasis (soluble form of E-selectin (sE-sel), soluble form of ICAM-1 (sICAM-1), von Willebrand factor (vWF), plasminogen activator inhibitor-1 (PAI-1), t-PA (tissue plasminogen activator); Figures 4F–J, respectively), and platelet activation (soluble form of P-selectin (sP-sel) and thrombospondin 1 (THBS-1); Figures 4K,L, respectively).

There were no significant changes in the plasma concentration of Ang-1 (Figure 4A), while plasma Ang-2 concentration and soluble form of Tie-2 receptor for angiopoetins were both increased by dabigatran treatment 2 days after the injection of breast cancer cells compared to untreated mice (Figure 4B). However, concentration of soluble form of sFLt-1 receptor for VEGF was lower in the plasma of dabigatran-treated mice 24 h after the injection compared to untreated mice (Figure 4D). Plasma concentration of SDC-1 was increased by dabigatran treatment 24 h after the injection of cancer cells, but this difference disappeared 2 days after cancer cells injection i.v. (Figure 4E). No changes were detected in the plasma sE-sel (Figure 4F), sICAM-1 (Figure 4G), vWF (Figure 4H), and PAI-1 (Figure 4I), while tPA-1 concentration in the plasma was decreased by dabigatran treatment 24 h after i.v. injection of cancer cells (Figure 4J). Concentrations of sP-sel (Figure 4K) and THBS-1 (Figure 4L) were lower in dabigatran-treated mice 2 days after injection of 4T1 breast cancer cells.

The most important changes between untreated and dabigatran-treated mice were detected 24 h and 2 days after i.v. injection of cancer cells. They corresponded to increased vascular permeability in the lungs 24 h after i.v. injection of cancer cells (enabling more efficient extravasation of cancer cells) what, consequently, induced more pronounced inflammatory response in the lungs of dabigatran-treated mice 2 days after i.v. injection of cancer cells. We claim that higher pulmonary endothelium permeability 24 h after i.v. and, consequently, higher levels of Ang-2 (Figure 3B), E-selectin (Figure 3C) and MMP-9 (Figure 3D) 2 days after i.v., provided more favourable microenvironemnt for the increased settlement of 4T1 breast cancer cells in the lungs of dabigatran-treated mice. The earliest plasma marker indicating more pronounced endothelial damage in dabigatran-treated mice was increased syndecan-1 (SDC-1), the proteoglycan of endothelial glycocalyx and determinant of blood vessel permeability (Fernández-Sarmiento et al., 2020) In fact, plasma SDC-1 was higher 24 h after i.v., indicating its increased shedding from endothelial surface into the circulation in dabigatran-treated mice at that time (Figure 4E) what was compatible with the alterations in lung endothelial permeability at that time. Interestingly, 2 days after i.v. injection of cancer cells, upregulation of Ang-2 (Figure 4B) and the soluble form of its receptor Tie-2 (Figure 4C) were detectable in the plasma, while the concentration of sFlt-1 in the plasma was not increased (Figure 4D), indicating that, in contrast to Ang-2-dependent pathway, VEGF-dependent signalling might be not involved in the negative effects of dabigatran in 4T1 breast cancer cell-injected mice. Finally, in dabigatran-treated mice 2 days after i.v., injection of cancer cells concentration of biomarkers of platelet activation was lower as judged by a lower THBS-1 (Figure 4L) and sP-sel (Figure 4K), underscoring platelet inhibition, what could have resulted in inefficient platelet-dependent protection of pulmonary endothelium integrity in inflammatory conditions (Middleton et al., 2016). Interesting early changes were also observed in the plasma concentrations of plasminogen activator (t-PA) that, surprisingly, was lower in dabigatran-treated mice 24 h after i.v. injection of cancer cells (Figure 4J) what might have facilitated pro-inflammatory activation of macrophages as shown by (Miles and Parmer, 2017).

It is worth noting that dabigatran treatment did not affect the concentration of abovementioned markers of endothelial dysfunction in plasma in healthy mice with the exception of vWF, the concentration of which was lowered (Supplementary Figure S1).

### 3.5 Effects of Dabigatran Treatment on Platelet Activation in BALB/c Mice Injected With 4T1 Breast Cancer Cells

**FIGURE 5:**
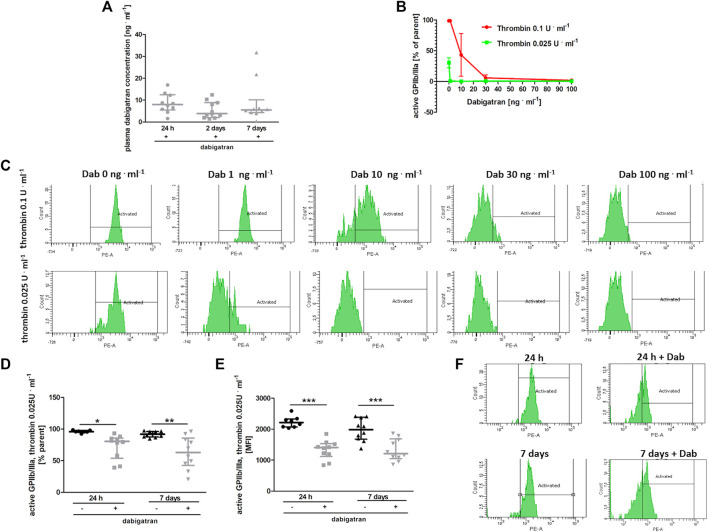
Effects of dabigatran on thrombin-induced platelet reactivity measured *ex vivo*. In **(A,D–F)**, mice were treated with vehicle (black symbols) or dabigatran etexilate (grey symbols) as described in Section 2.5, injected with 4T1 breast cancer cells, and euthanized at 24 h, 2 days, and 7 days after injection, whereas in **(B,C)**, washed blood samples of healthy mice were pre-incubated with dabigatran *ex vivo*. **(A)** shows the mean concentration of dabigatran in the animal plasma at the time of the euthanasia (approximately 6–8 h after the last oral dabigatran gavage). **(B)** shows the expression of an active form of GPIIb/IIIa on the surface of platelets in whole blood obtained from healthy animals. The blood samples isolated from these healthy animals were washed, pre-treated *ex vivo* with dabigatran at concentrations of 1, 10, 30, and 100 ng ml^−1^, and activated with bovine thrombin at a dose of .025 or .1 U ml^−1^. The data in **(B)** are presented as the mean and SEM of 2–3 independent experiments. Dabigatran alone at these concentrations did not affect the expression of the active form of GPIIb/IIIa (data not shown). **(C)** shows the representative flow cytometry results presented in **(B)**. In **(D)** and **(E)**, expression of an active form of GPIIb/IIIa on the platelet surface is shown as the percentage of the total (parent) platelet population and median fluorescence intensity (MFI) on the platelet surface, respectively, presented after activation of with .025 U ml^−1^ of bovine thrombin of washed blood samples obtained from untreated and dabigatran-treated 4T1 breast cancer cell-injected mice. In **(F)**, the representative flow cytometry data of **(D,E)** are shown. The data were analysed with one-way ANOVA **(A)** or two-way ANOVA **(D,E)** followed by appropriate *post hoc* test, and the results are presented as median ± IQR. The symbols *, **, and *** denote statistical significance at *p* < .05, *p* < .01, and *p* < .001, respectively.

At the time of blood collection, the plasma concentration of dabigatran was about 10 ng ⋅ ml^−1^ in all experimental groups (Figure 5A). To test whether such a concentration of dabigatran affected thrombin-induced platelet activation, platelets were stimulated *ex vivo* by low (.025 U ml^−1^) or high (.1 U ml^−1^) concentrations of thrombin in the absence or presence of dabigatran at a concentration range of 1–100 ng ⋅ ml^−1^, and the expression of active GpIIb/IIIa receptor was quantified by flow cytometry (Figures 5B,C). As shown in Figures 5B,C, even a 1 ng ⋅ ml^−1^ concentration of dabigatran inhibited platelet activation induced by .025 U ml^−1^ of thrombin. In turn, 30 ng ⋅ ml^−1^ dabigatran completely inhibited platelet activation induced by a higher concentration of thrombin (.1 U ml^−1^). Furthermore, the reactivity of platelets obtained from dabigatran-treated mice was diminished both at 24 h as well as on the 7^th^ day after i.v. injection of 4T1 breast cancer cells, as evidenced by diminished expression of the active form of GPIIb/IIIa in dabigatran-treated mice compared with non-treated mice in response to thrombin (.025 U ml^−1^) (Figures 5D–F), directly supporting the inhibitory effects of dabigatran treatment *in vivo* on thrombin-dependent platelet activation*.*

### 3.6 Effects of the Releasate From Thrombin-Stimulated Platelets on Pulmonary Endothelial Barrier Integrity in the Absence or Presence of Dabigatran

**FIGURE 6:**
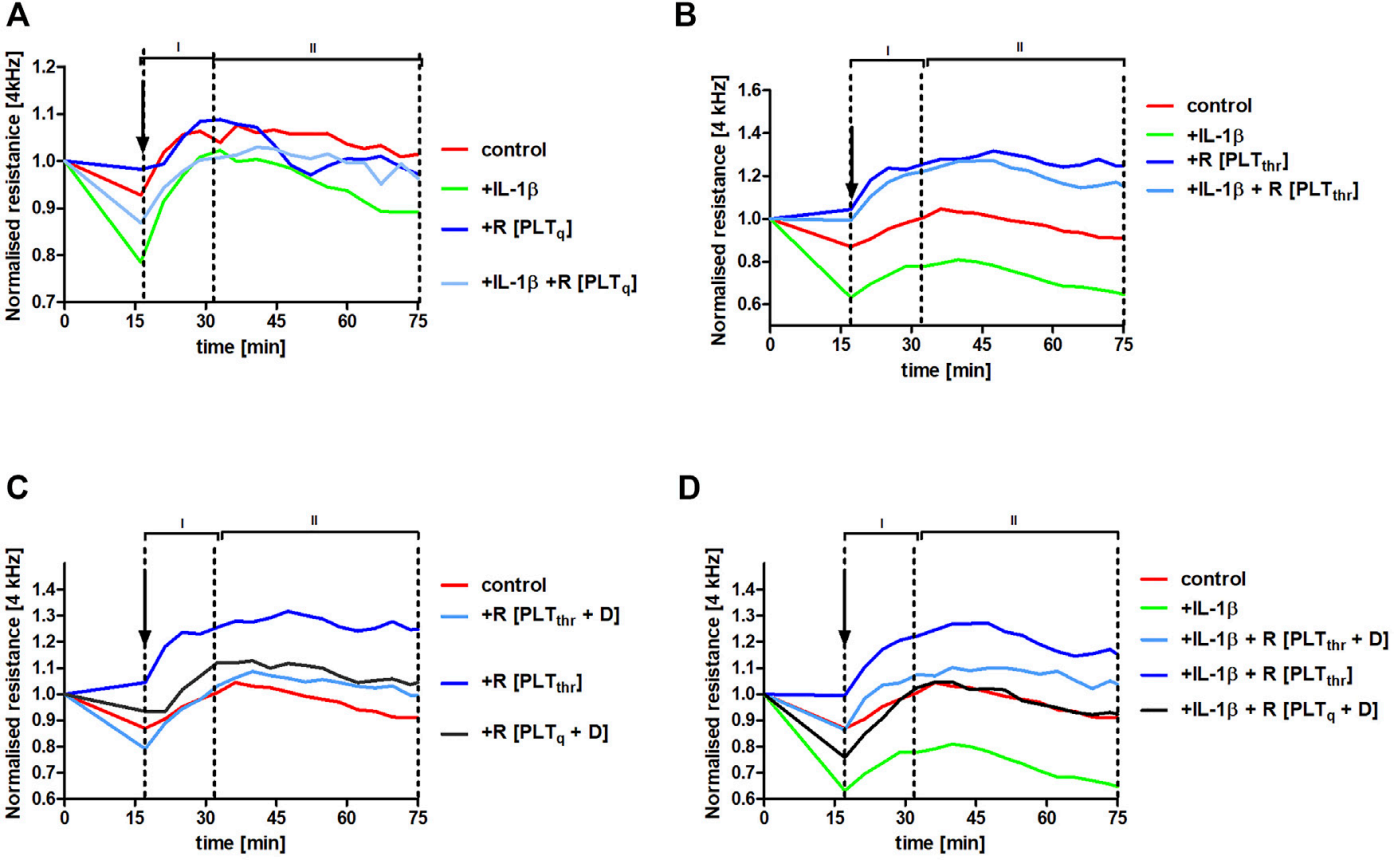
Protection of human pulmonary endothelial barrier compromised by IL-1β stimulation afforded by releasates of washed human platelets and the reversal of this effect by dabigatran. Human lung microvascular endothelial cell (HLMVEC) monolayers were treated with releasates derived from non-stimulated PLT (R [PLT_q_]) **(A)** or PLT stimulated with .1 U ml^−1^ thrombin (R [PLT_thr_]) **(B)** in the presence of 10 ng ml^−1^ IL-1β or without IL-1β. The effect of releasates derived from thrombin-stimulated PLT on the barrier integrity formed by HLMVECs in the presence of 30 ng ml^−1^ of dabigatran (R [PLT_thr_ + D]) was also tested without IL-1β **(C)** or in the presence of IL-1β **(D)**. The data are shown as the mean of 3–4 independent experiments and were analysed using analysis of covariance (ANCOVA) in the stable phase (II). Thrombin alone added in Tyrode buffer (.1 U ml^−1^; the final concentration of .01 U ml^−1^) did not affect endothelial barrier integrity.

To verify whether platelet releasate from thrombin-activated platelets could protect pulmonary endothelial barrier integrity in inflammatory conditions, HLMVECs monolayers were challenged with the inflammatory mediator interleukin (IL)-1β. This cytokine was chosen as it is released *in vivo* by activated monocytes/macrophages, increasing pulmonary permeability in the model of experimental metastasis (Häuselmann et al., 2016). IL-1β impaired endothelial barrier function (control vs. IL-1β, *p* < .001) (Figures 6A,B,D). However, in the presence of the releasate from washed non-stimulated (quiescent) human platelets R [PLT_q_] (Figure 6A) as well as in the presence of releasate of washed thrombin-stimulated human platelets R [PLT_thr_] (Figure 6B), IL-1β-induced impairment of endothelial barrier function was attenuated (*p* < .05 and *p* < .001, respectively), clearly showing platelet-dependent protection of the endothelial barrier in the lungs. The protective effect of platelet releasates afforded by thrombin-stimulated human platelets (*p* < .001) (Figure 6B) was more pronounced compared with non-stimulated platelets (*p* < .05) (Figure 6A). Most importantly, dabigatran substantially inhibited the pulmonary endothelium barrier-supportive effect of thrombin-stimulated platelet releasate (R [PLT_thr_ + D]) in the absence of IL-1β (Figure 6C) and in the presence of IL-1β (Figure 6D) (*p* < .001 and *p* < .001, respectively).

Furthermore, the barrier-protective effect of platelet releasates in the presence of IL-1β was to some degree agonist-specific (Figure 6; Supplementary Figure S2). Although thrombin- and collagen-stimulated PLT releasates protected the endothelial barrier integrity in the absence and in the presence of IL-1β, ADP-stimulated PLT releasate had the negative effect on the endothelial barrier in the absence (*p* < .001) as well as in the presence (*p* < .001) of IL-1β compared to control.

## 4 Discussion

In the present study, we demonstrated that dabigatran, a direct thrombin inhibitor, increased pulmonary metastasis in the hematogenous model of breast cancer metastasis in BALB/c mice. This effect was associated with higher pulmonary endothelial permeability *in vivo* and was ascribed to the inhibition of platelet-dependent protection of the pulmonary endothelial barrier during the inflammatory response alongside pulmonary metastasis. Indeed, using an *in vitro* assay based on impedance measurement, we confirmed that platelet activation by thrombin prevented the impairment of the pulmonary endothelial barrier induced by the pro-inflammatory cytokine IL-1β, and this protective effect was largely reversed in the presence of dabigatran. Altogether, our report suggests that inhibition of platelet-dependent protection of the pulmonary endothelial barrier in inflammatory conditions associated with cancer metastasis may, in fact, be detrimental and lead to increased endothelial permeability, endothelial activation, and endothelial inflammation, all of which favour metastatic spread.

Although we did not identify the molecule(s) responsible for platelet-dependent protection of the pulmonary endothelial barrier during cancer-associated inflammation, to our knowledge our study is the first to highlight the vital role of platelet-dependent protection of pulmonary endothelial barrier integrity in the setting of cancer metastasis. Our results may also explain some detrimental effects of antiplatelet therapy on cancer progression that have been described previously (Niers et al., 2009; Serebruany et al., 2015; Shi et al., 2017). Accordingly, we claim that the mechanisms of platelet-dependent regulation of inflammation-associated haemostasis (Ho-Tin-Noé et al., 2018) might determine the outcome of antiplatelet therapy on cancer metastasis (Smeda et al., 2020b).

Previously, an increased number of lung metastases after prolonged thrombin inhibition was observed by Niers et al. (2009). In that study, anti-coagulant (heparin or ximelagatran) treatment was terminated 24 h before cancer cell injection into mice. The authors ascribed their findings to increased cancer cell extravasation but did not provide any supporting experimental evidence in contrast to our study (Figure 3A). In our experiments, increased pulmonary metastasis (Figure 1) and lung permeability (Figure 3A) in dabigatran-treated mice were not timely linked to changes in thrombin activity expressed as a lag time to cleavage of fluorogenic substrate by free thrombin (Figure 2B). Similarly, there was no changes in the activation of the innate immune response as evidenced by fibrin deposition in the lungs (Charles et al., 2011) (Figure 2A), INFγ concentration (Figure 2C), and complement activation (Figure 2D) in dabigatran-treated mice. Yet, we provided evidence ascribing the detrimental effects of dabigatran to the inhibition of platelets, that are not only critical players finely tuning inflammatory responses of the host (Ribeiro et al., 2019) but also safeguard endothelial barrier integrity in inflammatory conditions (Ho-Tin-Noé et al., 2018).

At the time of the euthanasia, plasma dabigatran concentration in mice treated with this compound was quite low (Figure 5A), what could explain the lack of evident changes in coagulation measured in plasma based on CAT assay (Tchaikovski et al., 2007). Mice are efficient metabolisers of dabigatran (t½ = 1.25 h), and oral administration of the drug twice daily does not result in steady-state plasma levels in mice (DeFeo et al., 2010) as it does in humans (t½ = 8 h) (Blech et al., 2008). Nevertheless, 6–7 h after the last dabigatran dose administered by oral gavage, as in the studies by DeFeo et al. (2010) and Feinauer et al. (2020), the plasma dabigatran concentration was approximately 10 ng ⋅ ml^−1^ (Figure 5A). At this concentration, dabigatran inhibited thrombin-dependent platelet activation, as demonstrated in *ex vivo* (Figures 5D–F) and *in vitro* (Figures 5B,C) experiments. Dabigatran also dampened platelet-dependent protection of pulmonary endothelial barrier integrity *in vitro*, which was elicited by the releasate derived from thrombin-activated platelets (Figures 6C,D). These results suggested that inhibition of platelet-dependent mechanisms, protecting the integrity of the endothelial barrier during an inflammatory response by dabigatran, might have resulted in increased pulmonary permeability and, consequently, metastasis in dabigatran-treated and 4T1 breast cancer cell-injected mice with underlying mechanism of impaired platelet-dependent inflammation-associated haemostasis (Ho-Tin-Noé and Jadoui, 2018; Suraj et al., 2019b).

In fact, numerous reports have indicated an endothelial barrier-supportive function of platelets (Nachman and Rafii, 2008; Ho-Tin-Noé and Jadoui, 2018; Ho-Tin-Noé et al., 2021) that protect the vascular wall against excessive leukocyte-mediated inflammatory damage and subsequent bleeding (Ho-Tin-Noé and Jadoui, 2018) by involving the activation of the platelet CLEC-2-inflammatory/macrophage podoplanin signalling pathway (Lax et al., 2017). The number of other platelet-dependent endothelial barrier protective mechanisms have been also proposed involving production of platelet–derived trophic factors (e.g. VEGF, BDNF) (Nachman and Rafii, 2008), Ang-1 (Braun et al., 2020), sphingosine-1 phosphate (S1P), stromal cell derived factor 1α (SDF1α), transforming growth factor β1 (TGFβ1), and adenosine nucleotides (Kleinbongard et al., 2021). Importantly, platelet-dependent protection of the endothelial barrier was described for various inflammatory stimuli, including primary cancer (Lax et al., 2017; Ho-Tin-Noé et al., 2018), as well as in case of cardioprotection following ischemia/reperfusion (Kleinbongard et al., 2021). However, the importance of platelet-dependent protective mechanisms has not yet been confirmed in the context of local inflammation alongside pulmonary metastasis. In our study, we did not investigate the signalling pathways of non-classical haemostasis described in other inflammatory settings (Ho-Tin-Noé et al., 2008; Payne et al., 2017). However, we provide compelling evidence that mechanisms of inflammation-associated haemostasis could play a key role in hematogenous metastasis to the lungs, an organ especially protected by platelets during inflammatory conditions as discussed by Middleton et al. (2016).

In the present study, we showed that releasates from thrombin-activated platelets effectively protected the pulmonary endothelial barrier in the presence of inflammatory stimuli (IL-1β) *in vitro*, and dabigatran dampened this protective effect (Figure 6D). Furthermore, dabigatran at a concentration as low as 1 ng ⋅ ml^−1^ significantly inhibited platelet reactivity to a low dose of thrombin (.025 U ⋅ ml^−1^) (Figures 5B,C). When stimulation with .1 U ⋅ ml^−1^ of thrombin was used (this concentration of thrombin initiates platelet aggregation *ex vivo* (Chen et al., 2004)), inhibition of platelet reactivity to thrombin by dabigatran was detectable at a concentration as low as 10 ng ⋅ ml^−1^ (Figures 5B,C). Therefore, the inhibition of platelet reactivity to thrombin by low concentrations of dabigatran, which did not inhibit fibrin deposition in the lungs (Figure 2A) or thrombin generation in the plasma (Figure 2B), might have abrogated platelet-dependent mechanisms of pulmonary endothelial barrier protection. Thus, the endothelial barrier may have been rendered more prone to disruption in the inflammatory setting of cancer metastasis (Häuselmann et al., 2016) after dabigatran treatment what lead to increased lung permeability of dabigatran-treated mice (Figure 3A), as hypothesized recently by Ho-Tin-Noé et al. (2021). Indeed, thrombin-activated platelets were shown to release exosomes that inhibited the expression of adhesive molecules by endothelial cells (Li et al., 2017). It is worth noting that the protective effect of platelet releasate depended on the agonist used because thrombin- and collagen-stimulated platelet releasate produced the protective effects, while releasate derived from ADP-stimulated platelets did not restore endothelium integrity in the presence of an inflammatory agent (Figures 6; Supplementary Figure S1), as shown previously (Patil et al., 1997).

Accordingly, the results presented in this work might explain some recent clinical (Serebruany et al., 2015; McNeil et al., 2018) and experimental (Niers et al., 2009; Smeda et al., 2020a) data showing cancer-promoting effects of anticoagulant/antiplatelet treatment in humans and animal models, respectively, and also are in line with the upcoming controversies regarding the use of agents affecting platelet reactivity in cancer treatment (Wojtukiewicz et al., 2017; Smeda et al., 2020b; Najidh et al., 2020).

On the other hand, our data showing an increased number of pulmonary metastases after dabigatran treatment (Figure 1) are in opposition with the reports of DeFeo et al. (2010) and Feinauer et al. (2020), where dabigatran administered in a similar regimen as in our work effectively reduced metastatic spread in the liver and brain, respectively. However, dabigatran treatment did not reduce lung metastasis, started neither prior to orthotopic injection of 4T1 breast cancer cells (DeFeo et al., 2010), nor started after orthotopic injection of 4T1 breast cancer cells when the primary tumours were already palpable (Alexander et al., 2015). Accordingly, the abovementioned discrepancies may be partially ascribed to the fact that the effect of dabigatran on primary tumour may be linked to distinct mechanisms as compared with the mechanism proposed here, pertaining to the extravasation and metastasis in the pulmonary circulation and, thus, representing possibly another example of the mechanistically distinct response to the same pharmacological intervention for primary tumour and metastatic cancer cells as we showed previously (Kij et al., 2018). Indeed, dabigatran was shown to antagonize growth, cell-cycle progression, migration, and endothelial tube formation induced by thrombin in the invasive breast cancer cell line expressing high levels of protease-activated receptor 1 (PAR-1) (Vianello et al., 2016) that was, most probably, the result of p53 mutation (Salah et al., 2008). However, at the same time dabigatran could antagonise PAR receptors on platelets. Furthermore, endothelium-protective activity of platelets might be organ-specific with particular importance in the lungs rather than in the liver as thrombocytopenia significantly increased pulmonary vascular permeability, an effect reversed by restoring the circulating platelet population as shown by Lo et al. (1988).

Altogether, our work points out that efficient targeting of platelets to combat cancer requires a better understanding of the platelet–dependent mechanisms involved in the preservation of the pulmonary endothelial barrier as compared with those of platelet–cancer cell interactions favouring metastasis in order to target the latter mechanisms selectively (Smeda et al., 2020b). Of note, the effect described in our work, demonstrating that the impairment of the protective function of platelets would lead to the increased endothelial permeability and more efficient 4T1 breast cancer cell extravasation at the metastatic site, does not seem to be a cancer-type specific response since Niers et al. (2009) showed the same effect in case of prolonged thrombin inhibition in the murine model of melanoma.

Therefore, to develop effective antimetastatic strategies based on platelet inhibition, more research is necessary to elucidate the complex role of platelets in the metastatic process and platelet-dependent protection of endothelial barrier alongside cancer progression and dissemination. Indeed, the role of platelets in cancer seems to go far beyond the already well-known mechanisms favouring cancer seeding promoted by platelet aggregation and thrombus formation and also encompasses platelet-dependent mechanisms regulating endothelial barrier function.

## Data Availability

The raw data supporting the conclusion of this article will be made available by the authors, without undue reservation.
