# Supplementary material for: Direct Thrombin Inhibitor Dabigatran Compromises Pulmonary Endothelial Integrity in a Murine Model of Breast Cancer Metastasis to the Lungs; the Role of Platelets and Inflammation-Associated Haemostasis
Source: Front Pharmacol. 2022 Feb 28;13:834472. doi: 10.3389/fphar.2022.834472 (PMC8918823; doi:10.3389/fphar.2022.834472)
Supplement: Supplementary file 1 [file DataSheet1.docx]

Supplementary Material

## 1 Supplementary Figures


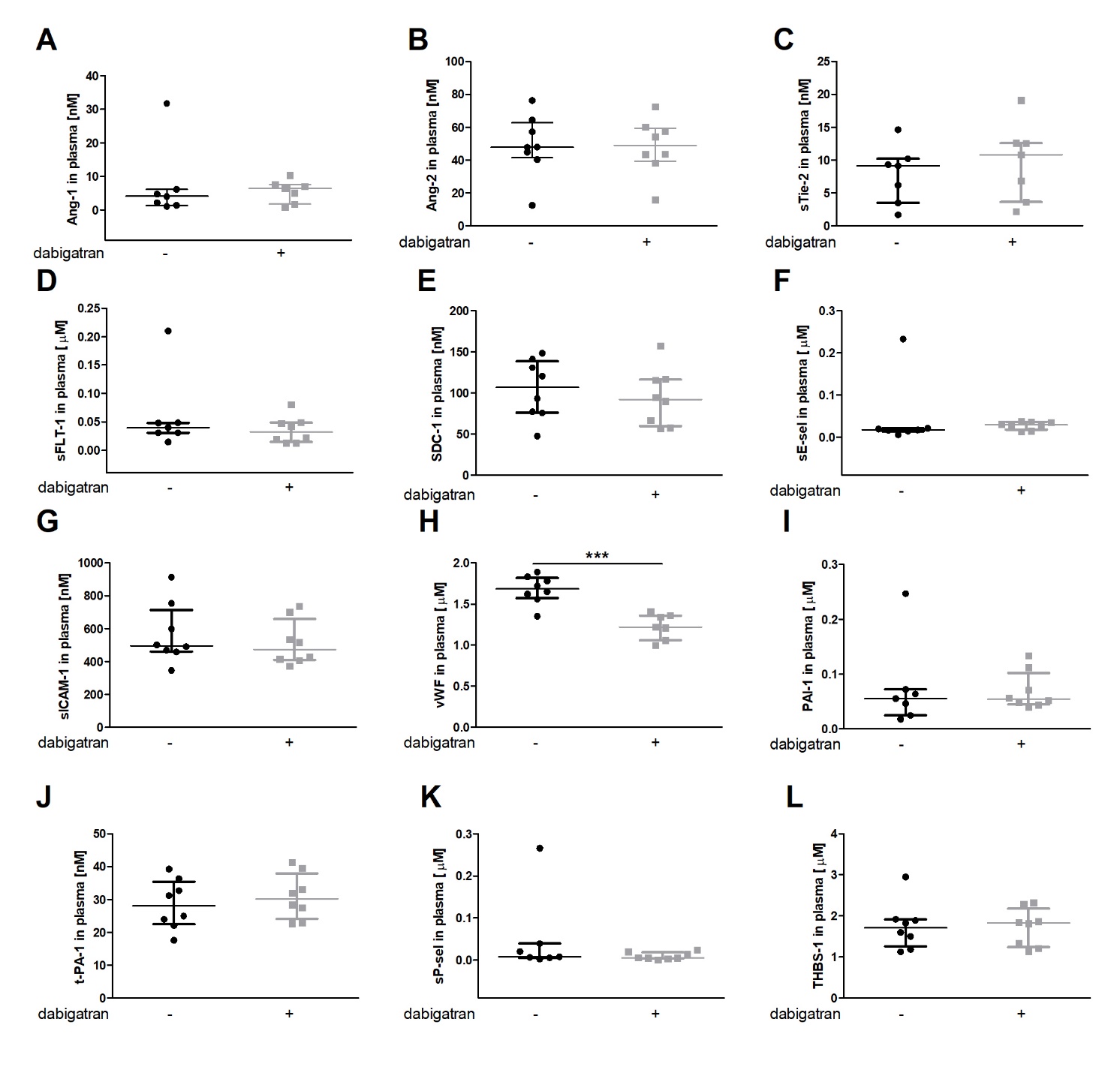


**Supplementary Figure 1. Effects of dabigatran etexilate treatment on the panel of plasma biomarkers of endothelial dysfunction in healthy mice**. Dabigatran etexilate treatment was administered for 2.5 days twice daily by oral gavage (first dose: 150 mg per kg of body weight, later doses: 100 mg per kg of body weight) in 0.05% natrosol (healthy dabigatran-treated mice; grey symbols) while the control mice received only the vehicle (healthy untreated mice; black symbols). The panel of selected biomarkers of endothelial dysfunction (Ang-1 (**A**), Ang-2 (**B**), sTie-2 (**C**), sFLT-1 (**D**), SDC-1 (**E**), sE-sel (**F**), sICAM-1 (**G**), vWF (**H**), PAI-1 (**I**), t-PA (**J**), sP-sel (**K**), and THBS-1 (**L**)) was measured in the plasma using the microLC/MS-MRM method as described in the main manuscript body. The data are presented as the median ± IQR. Statistical analysis was performed with parametric unpaired Student t-test **(B, C, E, G, H, J, L**) or non-parametric Mann-Whitney test (**A, D, F, I, K**) based on normality of data distribution measured with Shapiro-Wilk normality test. The outliers identified by Grubbs test were excluded from the analysis. The symbol *** denotes statistical significance at P < 0.001.


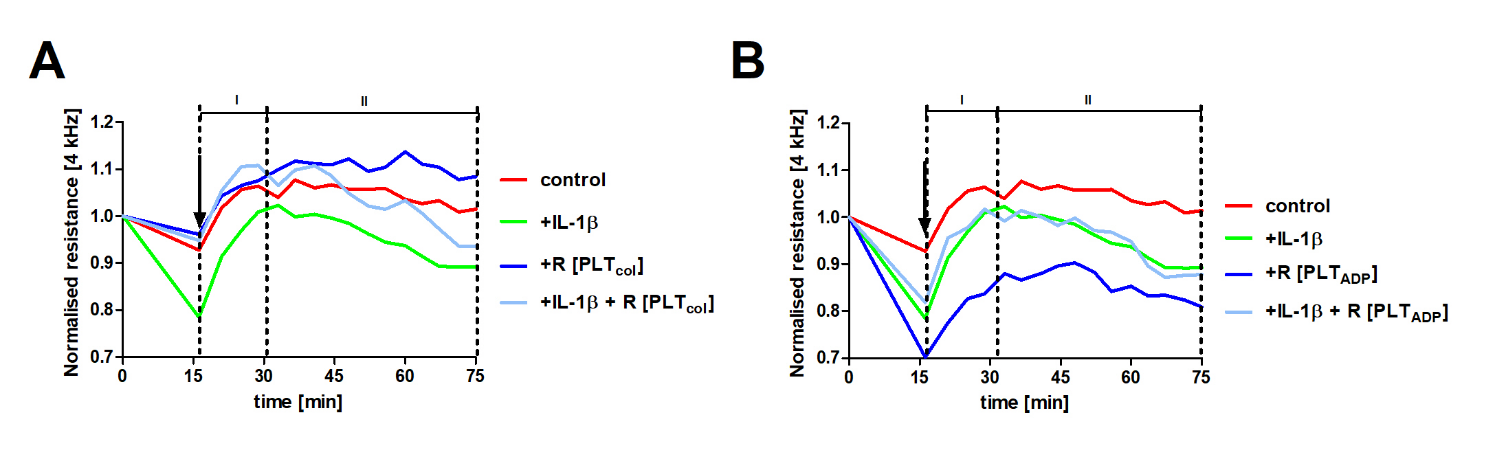


**Supplementary Figure 2. Effects of collagen- and ADP-stimulated platelet releasates on the human pulmonary endothelial barrier *in vitro***. Human lung microvascular endothelial cell monolayers were treated with washed PLT releasates after PLT stimulation with (**A**) collagen (5 μg ⋅ ml^-1^) (R [PLT_col_]) and (**B**) ADP (20 μM) (R [PLT_ADP_]) in the presence of IL-1β (10 ng ⋅ ml^-1^) or without IL-1β. The data are shown as the mean of 3–4 independent experiments and were analysed using analysis of covariance (ANCOVA) in the late stable phase (II) using Statistica 13.
